# Multivessel Coronary Artery Disease Presenting as a False-Negative Nuclear Stress Test: A Case of Balanced Ischemia

**DOI:** 10.7759/cureus.53789

**Published:** 2024-02-07

**Authors:** Allan E Santos Argueta, Junaid Ali, Asim Khan, Birgurman Singh, Dinesh Singal

**Affiliations:** 1 Internal Medicine, Saint Peter’s University Hospital, New Brunswick, USA; 2 Cardiology, Saint Peter’s University Hospital, New Brunswick, USA

**Keywords:** cad: coronary artery disease, false negative nuclear stress test, balanced ischemia, nuclear stress myocardial perfusion imaging, multivessel coronary artery disease (mvcad)

## Abstract

Myocardial perfusion imaging (MPI) is fundamental to comparing coronary vessel perfusion levels and guides in identifying ischemic areas. However, false negatives, such as balanced ischemia, are important considerations in interpreting these results. In this case report, we describe a 77-year-old female who presented with cardiac chest pain with normal laboratory results, electrocardiogram, and imaging. However, given her history and risk factors, left heart catheterization was performed, which showed triple vessel coronary artery disease.

## Introduction

Cardiovascular disease is the leading cause of death worldwide. Myocardial perfusion imaging (MPI) is a reliable diagnostic tool for assessing myocardial perfusion in the diagnosis and risk stratification of coronary artery disease (CAD) [1]. However, interpreting MPI is challenging since images depend on the myocardial region with the relatively highest radiotracer uptake based on perfusion at rest and under stress [2,3]. Approximately 10-20% of MPI results are false negatives (FN) due to factors such as branch vessel stenosis, left circumflex artery stenosis, inadequate exercise, caffeine intake, and balanced ischemia [3-5]. Patients with normal MPI results are rarely referred for catheterization [6]. In this report, we present a rare case of coronary balanced ischemia identified through MPI, with multivessel CAD confirmed by an invasive coronary angiogram.

## Case presentation

**Figure 1:**
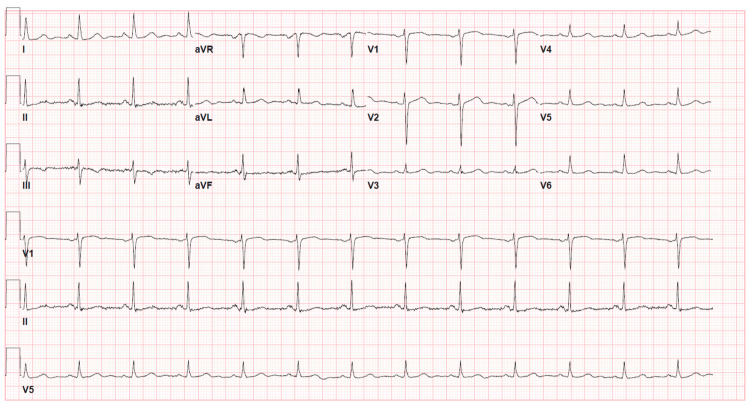
Resting electrocardiogram on admission The electrocardiogram shows a normal sinus rhythm with no acute ischemic changes.

A 77-year-old female with hypertension, type 2 diabetes mellitus, and hyperlipidemia presented to the hospital within one hour of experiencing mid-sternal chest pain that radiated to both arms. She described the pain as dull, lasting for about 10 minutes, with no alleviating or exacerbating factors. It was associated with a sense of impending doom, profuse sweating, lightheadedness, and transient blurriness of vision. Despite the complete resolution of symptoms, the patient decided to seek medical attention, reporting that she had never experienced similar symptoms before and was frightened by the sensation she felt. Upon examination, her blood pressure was 171/70 mmHg, heart rate was 74 beats per minute, and SpO2 was 98% on room air. The physical examination was unremarkable. An electrocardiogram (EKG) showed normal sinus rhythm with no acute changes (Figure 1). Chest X-ray and her initial blood workup, including a troponin level of <0.03 ng/mL, were unremarkable. Risk stratification was low according to both the Thrombolysis In Myocardial Infarction (TIMI) and the Global Registry of Acute Coronary Events (GRACE) scores.

**Figure 2:**
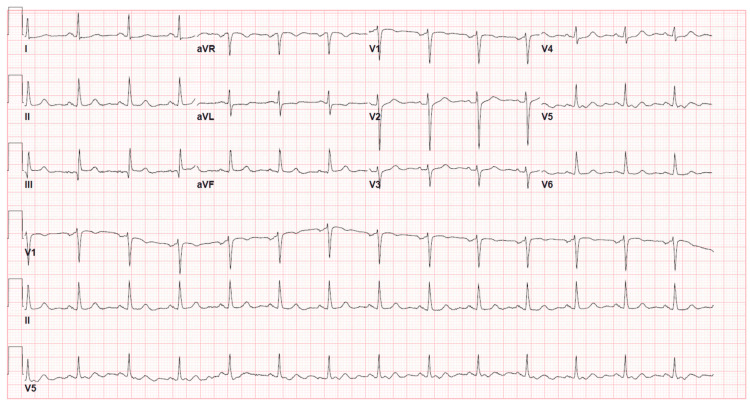
Repeated resting electrocardiogram Non-ischemic resting electrocardiogram.

**Figure 3:**
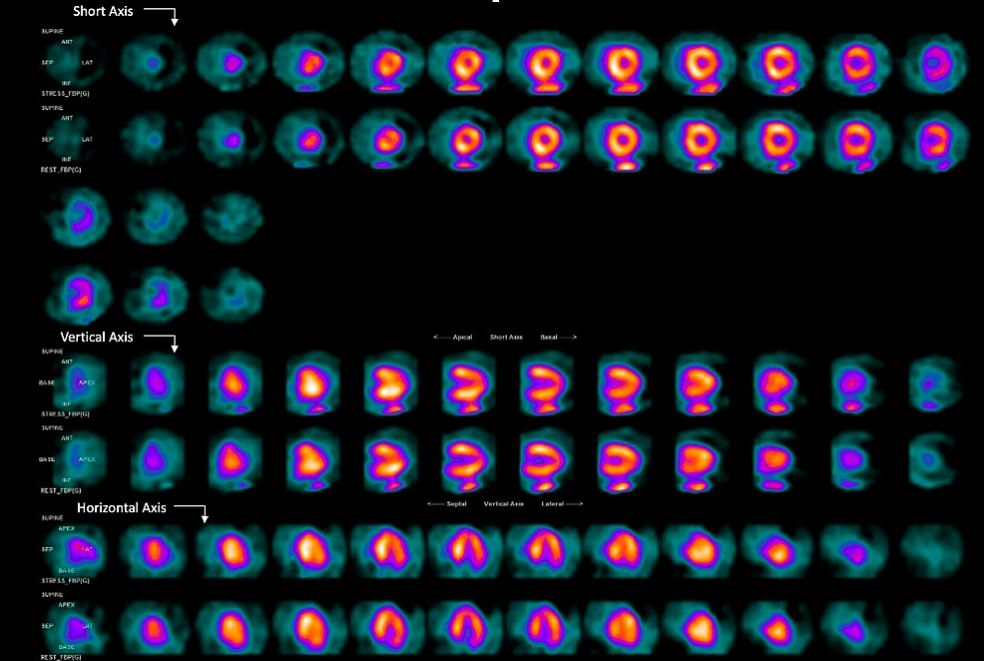
Myocardial perfusion imaging Non-ischemic SPECT (single-photon emission computed tomography) scan.

The patient was admitted for evaluation of acute coronary syndrome (ACS). At six hours, a repeat EKG was unchanged (Figure 2), and her troponin level slightly increased to 0.038 ng/mL, further rising to 0.09 ng/mL after 12 hours. The echocardiogram revealed a preserved ejection fraction with no wall hypokinesia. Subsequently, she underwent an adenosine nuclear stress test, which yielded non-ischemic stress EKG and MPI results (Figure 3).

**Figure 4:**
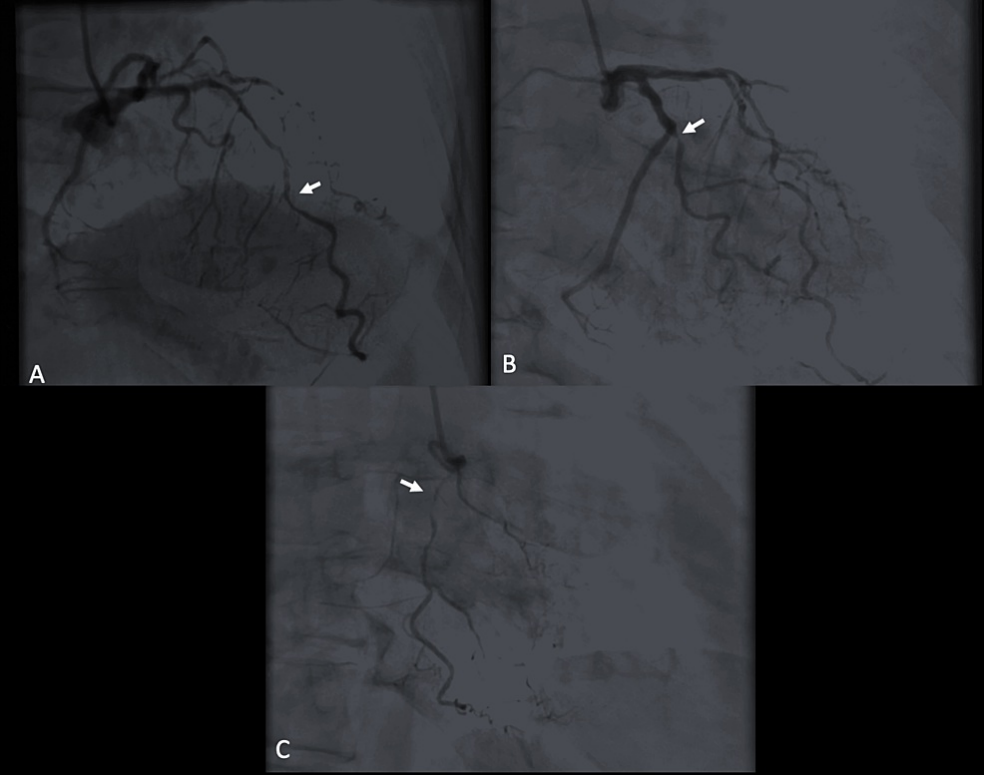
Angiographic pictures of left heart catheterization. (A) Mid-LAD artery luminal stenosis. (B) Severe proximal OM artery occlusion. (C) Severe proximal RCA lesion LAD: left anterior descending, OM: obtuse marginal, RCA: right coronary artery.

After undergoing the stress test, she experienced an episode slightly similar to the symptoms she initially had before her admission. Again, her EKG was unremarkable; however, her troponin level increased to 0.229 ng/mL. She underwent left heart catheterization within 24 hours, which revealed severe triple vessel disease (Figure 4). Consequently, coronary artery bypass grafting (CABG) was performed.

## Discussion

In this case report, we presented a patient with cardiac chest pain who had normal EKGs, cardiac markers, and non-invasive imaging but was found to have three-vessel CAD during a coronary angiogram.

MPI with either single photon emission computed tomography (SPECT) or positron emission tomography (PET) is widely used for the evaluation of CAD in patients with intermediate pre-test probability. However, despite MPI being reported as one of the most sensitive non-invasive tests for CAD, approximately 10-20% of MPI results are false negatives (FN) due to factors such as branch vessel stenosis, left circumflex artery stenosis, inadequate exercise, caffeine intake, and balanced ischemia [3-5].

Balanced ischemia, a rare cause of FN MPI, has a prevalence of 16%. It is characterized by a homogeneous decrease in blood flow through the coronary arteries, leading to an underestimation of CAD because of the balanced reduction in myocardial perfusion. This results in the radiotracer showing equal uptake in all portions of the myocardium noted on imaging [2,7,8]. Possible mechanisms include endothelial dysfunction, the presence of collateral circulation, global transmyocardial or subendocardial ischemia, and shifts in the endocardial-epicardial flow ratio [9,10].

Alternative diagnostic tools such as cardiac CT with coronary angiography with fractional flow reserve (FFR) or cardiac magnetic resonance (CMR) have their own limitations [5,11]. CT with FFR depends on high-quality images requiring a low heart rate, whereas MPI does not have these limitations. A significant limitation in measuring FFR is the assessment of epicardial vessels, while MPI assesses the entire vasculature [12]. Stress CMR has been shown to be another alternative in evaluating stable ischemic heart disease and could be considered superior to MPI; however, its limitations include the lack of combination with exercise, patient-related factors such as obesity and claustrophobia, and the use of ferromagnetic materials like implantable devices [13]. CT with FFR and CMR are emerging in their application for assessing CAD.

Given that MPI may underestimate high-risk CAD in selected patients, leading to a false negative result [14], a combination of clinical, functional, and MPI factors is essential to predict the high risk of CAD [15].

## Conclusions

History-taking remains the most critical element in evaluating patients for ACS. Balanced ischemia is a significant factor in interpreting MPI, as the global hypoperfusion in all the myocardium segments can be interpreted as non-ischemic. If missed, FN cases can have devastating outcomes.
